# Excess Lifetime Cancer Risk Associated with Granite Bearing Radioactive Minerals and Valuable Metals, Monqul Area, North Eastern Desert, Egypt

**DOI:** 10.3390/ma15124307

**Published:** 2022-06-17

**Authors:** Ahmed E. Abdel Gawad, Khaled G. Ali, Adel A. Abdel Wahed, Khalid Alsafi, Mawya Khafaji, Sarah Albahiti, Magdy Khalil, Masoud S. Masoud, Mohamed Y. Hanfi

**Affiliations:** 1 Nuclear Materials Authority, P.O. Box 530, El-Maadi, Cairo 11728, Egypt; khaled_ali@yahoo.com (K.G.A.); ahm.elsay@hotmail.com (A.A.A.W.); masoudsalah85@gmail.com (M.S.M.); 2 Medical Physics Unit, Department of Radiology, Faculty of Medicine, King Abdulaziz University (KAU), Jeddah 22252, Saudi Arabia; kalsafi@kau.edu.sa (K.A.); mkhafaji@kau.edu.sa (M.K.); shagi@kau.edu.sa (S.A.); 3 Medical Physics Unit, Diagnostic Imaging Department, King Abdulaziz University Hospital, Jeddah 22252, Saudi Arabia; 4 Geology Department, Faculty of Science, Damietta University, Damietta 34511, Egypt; mmkhalil50@yahoo.com; 5 Institute of Physics and Technology, Ural Federal University, 620002 Ekaterinburg, Russia

**Keywords:** monzogranites, spectroscopy, radioactivity, hazard effects, valuable metals, radioactive-bearing minerals

## Abstract

The present work is concerned with assessing the cancer risk contributed by the studied granite types including valuable metals, such as Cu, Au, and Ba mineralization, as well as radioactive-bearing mineralization, such as monazite and zircon, in south Monqul at Wadi Makhrag El Ebel, north Eastern Desert, Egypt. The mineralization analyses illustrated that copper mineralization containing chrysocolla and tenorite minerals were restricted to the alteration zone, especially (argillic, phyllic, and propylitic) in monzogranite. However, barite veinlets had an ENE–WSW trend, while gold mineralization was confined to quartz veins having NE–SW trends. Monazite and zircon are radioactive-bearing minerals recorded in monzogranite causing high radioactive zones in south Monqul. The radionuclide activity concentrations were detected in the studied monzogranites. The mean values of A_U_ (103 ± 91 Bq kg^−1^), A_Th_ (78 ± 19 Bq kg^−1^), and A_K_ (1484 ± 334 Bq kg^−1^) in the monzogranite samples were higher than the recommended worldwide average. The change in radioactive-transporting minerals found inside granite faults caused the high amounts of radioactivity seen in the samples. Due to the monzogranites being applied in building materials, the radiological hazards were assessed by calculating risk indices such as annual effective dose (AED) and excess lifetime cancer risk (ELCR). The acceptable limit for the ELCR readings was surpassed. As a result, the investigated monzogranite samples are not suitable for use in infrastructure materials.

## 1. Introduction

Granite is igneous rock composed primarily of quartz, K-feldspar, and mica that is utilized for decorative purposes both inside and outside the home, such as building and ornamental materials. These rocks carry radionuclides by their very nature. The most important sources of natural radioactivity in the environment are terrestrial and cosmic radiation. Ionizing radiation in naturally occurring radioactive elements (NORMs) makes the human body uneasy. External strategy exposure from the emitted gamma rays of terrestrial radionuclides such as ^238^U, ^232^Th, and ^40^K and internal mechanism exposure from breathed radon gas and its daughters are the two main forms of exposure [1,2,3]. 

In recent years, numerous evaluations of high natural background zones around the world have gained increased attention for risk assessment due to whole-body exposures to long-term, low-level radiation of populations. Previous research has shown that high radiation levels are caused by the presence of radionuclides in high concentrations in granite rocks, sediments, and soils, among other things. Granite and sediments, among other geological items, serve an important role in building materials and other infrastructure applications, as well as accumulating and transporting radionuclides from one zone to another [4,5]. As a result, numerous researchers have been able to distinguish between national surveys conducted around the world in recent decades for effectively detecting natural radionuclides in granite and sediments [6,7]. Background radiation in the environment is caused by terrestrial radionuclides and their daughters, as well as by cosmic radiation. Its existence in the environment is influenced by mineralogy, geochemistry, and physicochemistry [8,9]. 

Furthermore, the general population’s radiological impact is a prominent research area in radioecology, where data provide crucial and necessary information in the monitoring environmental contamination, allowing the public to receive more appropriate and effective protective recommendations [10,11]. To protect humans from gamma radiation, which can be generated by a variety of disorders, the emission of gamma radiation from radioactive materials must be continuously monitored [12,13]. Long-term radioactive exposure causes serious illnesses, such as oral necrosis, chronic lung disease, leukopenia, and anemia, according to the ATSDR (Agency for Toxic Substances and Disease Registry) [14,15]. Several studies have been conducted to determine the radiation danger and annual dose supply of natural radioactivity in construction materials [16,17]. Implementing a radiological environmental assessment for construction materials in order to study and regulate radioactive consequences on people and the environment is a significant and complex task that must be completed to achieve sustainable development objectives. Quantifiable variables that can be used as input parameters for modelling environmental distribution and estimating radiation dosage should be used to assess radiation impacts [18,19].

**Figure 1:**
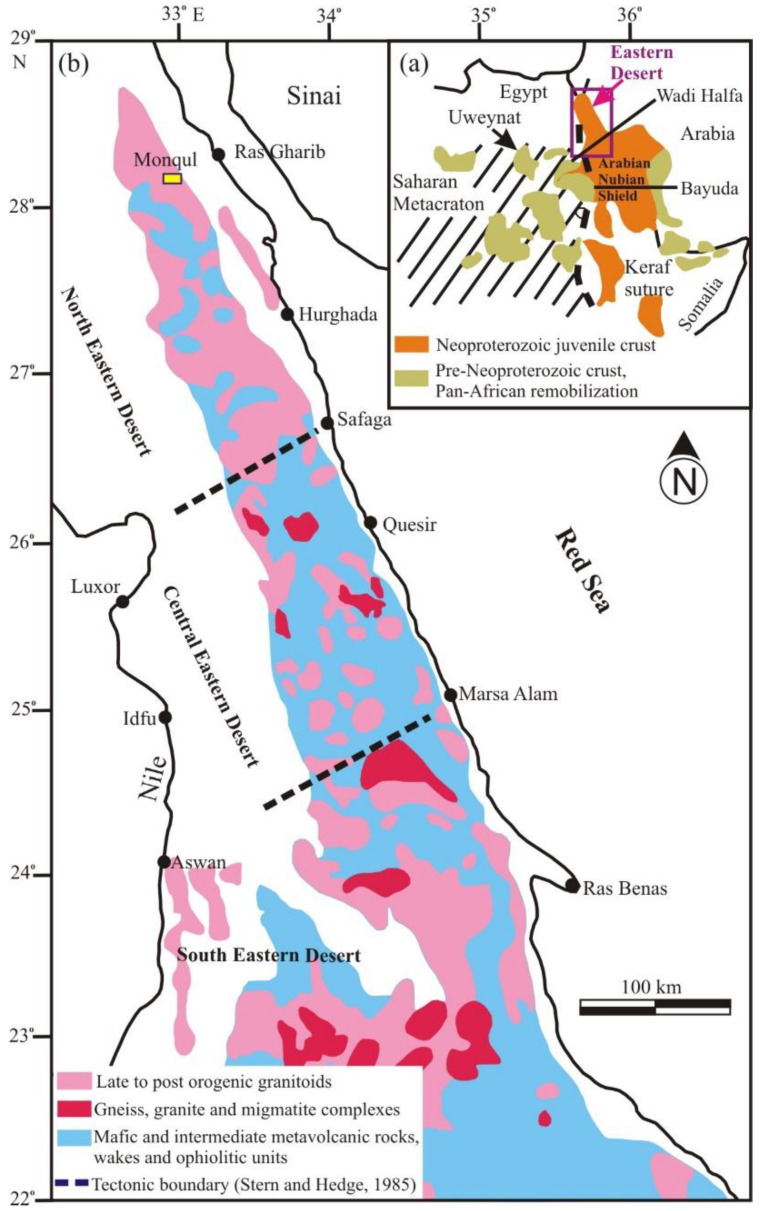
(**a**) Geologic map showing the Arabian–Nubian Shield (ANS); (**b**) geologic map showing the basement rocks of the Neoproterozoic age in the ED of Egypt [20].

The Eastern Desert (ED) massif of Egypt is considered a part of the Arabian–Nubian Shield (ANS) that was formed around the end of the Precambrian time. These rocks are well-exposed all over the Red Sea Hills in Egypt, Sudan, Ethiopia, Eritrea, Somalia, Saudi Arabia, Jordan, Oman, and Yemen [20] (Figure 1a,b). They are characterized by high exposure and a mountainous relief.

The East African Orogen (EAO) hosts many sites of gold mineralization. Gold has a strong exploration program, especially in Egypt, Tanzania, Malawi, Eritrea, Ethiopia, Kenya, and Mozambique [21]. Gold production in the ANS has a long historical record that was abandoned for a long time. There have been recent discoveries of high-tonnage, intrusion-related gold deposits, e.g., Sukari and Fawakhir–El Sid in Egypt, Ad Duwayhi in Arabia, Tulu Kapi in Ethiopia, and Koka in Eritrea [21]. The ED is considered one of the largest gold provinces both in the ANS and worldwide, with explorations of more than 200 gold sites [21,22,23,24].

It has been recognized that granite, related to pegmatite and acidic volcanics, is a good potential source of uranium, thorium, and other rare metal mineralization [25,26,27,28,29,30,31,32,33,34]. In the last ten years, comprehensive programs for U and Th exploration in the so-called younger granites (post-orogenic granites) of the ED have been carried out. These programs have led to the investigation of many sites of these granites bearing U, Th, Zr, Nb, Ta, Sn, and another valuable rare metals. Rare metal mineralization is restricted to granites such as the El Missikat [35,36], El-Erediya [37,38], Ras Abda [39,40,41], Gattar [42,43,44,45], and El Sela younger granite plutons in the south of the Eastern Desert [46,47,48,49]. The post-orogenic magmatism in the ED occurred between 530–620 Ma [50,51,52,53,54].

It is well known that porphyry copper mineralization is derived largely from the fractionation process associated with igneous rocks of calc-alkaline affinities [55,56]. These granites are regarded as good resources of magmatic fluids and heat for the formation of Cu-porphyry [57,58,59,60,61]. Copper plays a role of great importance in many industries related to high ductility, malleability, electronics, architecture, plumbing, building construction, thermal conductivity, and electrical and corrosion resistance.

The novelty of this study is that it evaluates the radioactive risk of using monzogranites in construction materials and infrastructure applications. As a result, the assessment is based on determining the public’s exposure to ionizing radiation emitted by radionuclides (^238^U, ^232^Th, and ^40^K) contained in the granites. Various radioactive hazard factors are calculated, including radium equivalent activity (Ra_eq_), absorbed dose rate (D_air_), and annual effective dose (AED). In addition, the effective dose due to the organs (D_o_) and the excess lifetime cancer risk (ELCR) are calculated.

## 2. Geologic Setting

**Figure 2:**
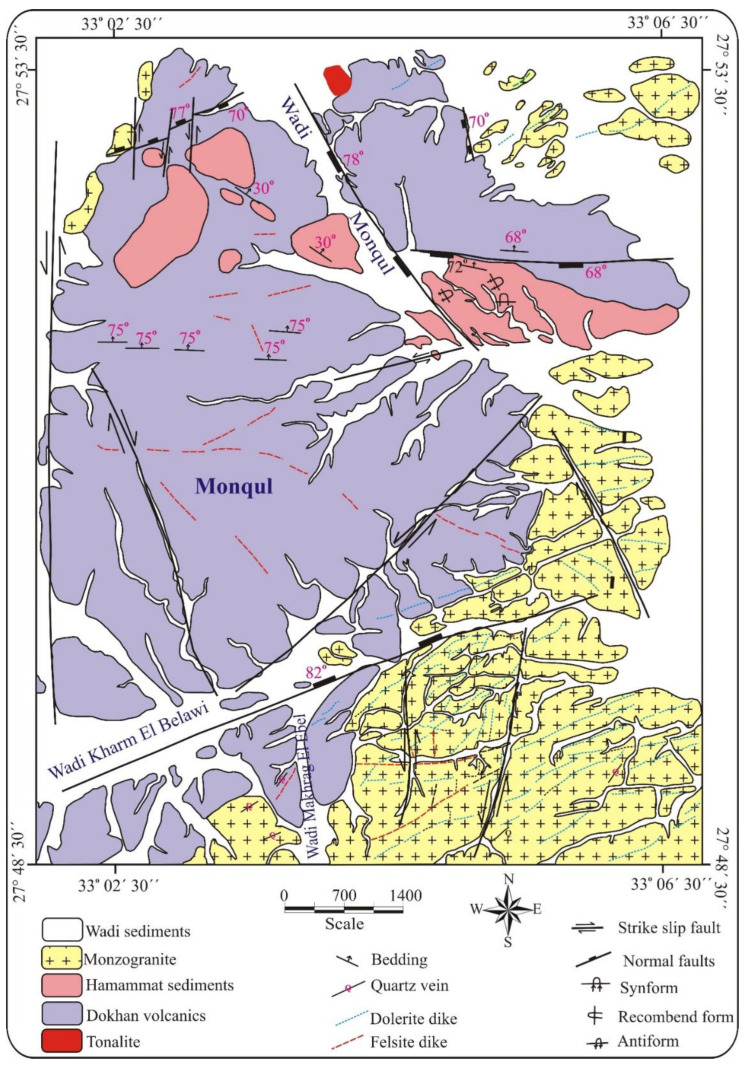
Geologic map of Monqul, north Eastern Desert of Egypt.

The Monqul area lies in the northern part of the ED (Figure 1b). A field investigation of the Monqul area showed that the exposed rock units are represented by older granitoids (tonalite-granodiorite), Dokhan volcanics, Hammamat sediments, and younger granite (monzogranite) dissected by dolerite and felsite dikes, as well as quartz veins (Figure 2). 

Older granitoids (tonalite-granodiorite) occur as small, scattered masses in the northern part of the mapped area. They are characterized by low-to-moderate relief and medium-to-coarse grains, and they are fractured, weathered, and vary in colors from whitish-grey to a greenish-grey, as well as porphyritic in texture. They are composed mainly of plagioclase quartz and mafic minerals and are set in a relatively finer groundmass.

Dokhan volcanics are mainly composed of stratified lava flows interbedded with subordinate pyroclastics. They consist of thick flows of andesite and, to a lesser extent, of basalt, and they range from buff to reddish colors of rhyolite, dacite, and rhyodacite. These rocks are hard, massive, fine-grained, and vary in color from whitish to reddish-pink, buff, pale red, and red, as well as dark green to grey and black. Pyroclastics are well-characterized by bands and include ash tuffs, lapilli tuffs, and lithic tuffs, as well as ignimbrite and imperial porphyries. 

**Figure 3:**
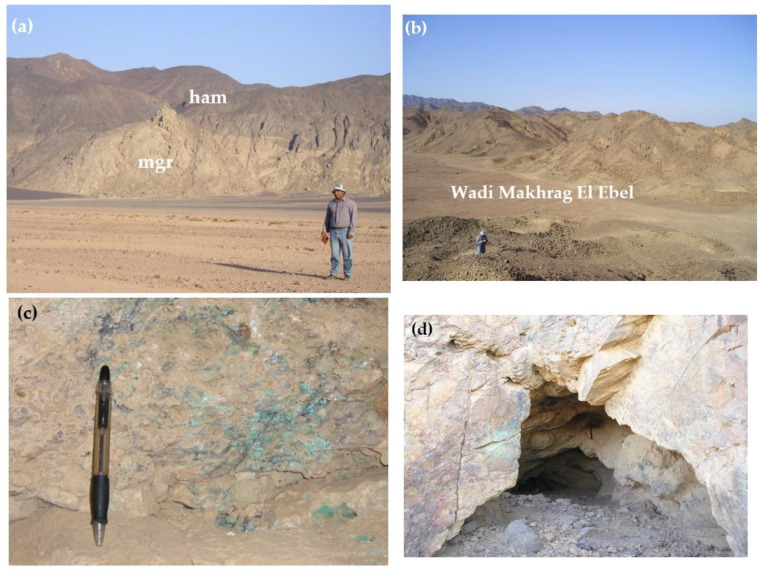

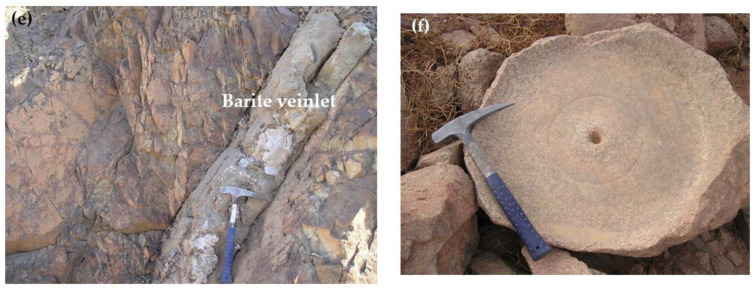
(**a**) Monzogranite (mgr) was intruded by Hammamat sediments (ham). (**b**) Alteration zones of Wadi Makhrag El Ebel bearing Cu, Au, and Ba mineralization. (**c**) Monzogranite-enclosed copper mineralization in south Monqul, Wadi Makhrag El Ebel. (**d**) Copper mineralization extracted from the area along the strike of the intersection between two conjugate normal fault systems in monzogranites trending E–W. (**e**) Barite-veinlet-dissected acidic volcanics having ENE–WSW trends. (**f**) Manual mill from monzogranite used for grinding quartz bearing gold.

The studied Dokhan volcanics were nonconformably followed upward by thick sequences of Hammamat sediments. Hammamat sediments are composed mainly of conglomerates, sandstone greywacke, and siltstone and are intruded by monzogranite (Figure 3a).

The Dokhan volcanics in the studied area intruded on granitoids with sharp contact. The studied rocks were extruded by Dokhan volcanics with sharp contact and were intruded by younger granite.

The younger granites are well-represented in the southern and northern parts of the studied area (Figure 2). They have low-to-moderate reliefs and are pale pink and coarse-grained with a porphyritic texture. They were subrounded by rounded mafic xenoliths of different sizes. These rocks were dissected by felsite and dolerite dikes, as well as by quartz and barite veins having N–S and E–W trends, respectively. They were affected by intensive alterations, including potassic, phyllic, argillic, and prophylitic types. The alteration zones were enriched in copper, barite, and gold mineralization in the southern part of the Monqul area at Wadi Makhrag El-Ebel (Figure 3b–f).

## 3. Materials and Methods

### 3.1. GS-256 Spectrometer

Ground gamma ray spectrometric measurements were conducted on Monqul monzogranites using a Geophysica Brno GS-256 spectrometer. The instrument is manufactured by Radiation Solution Inc in Sugar City, ID, USA. It had a 0.35 L sodium iodide (NaI) thallium-activated detector. A measuring interval of 120 s was maintained in order to allow sufficient time to establish a stable spectrum. The ground spectrometry survey sites were selected to cover granitoid exposures in the study area. The radioelement measurements were obtained using a single measurement from each site for equivalent thorium (eTh, ppm), equivalent uranium (eU, ppm), and potassium percent (K, %). The GS integrated a horizontal area of about 1 m diameter with nearly 25 cm of depth when in direct contact with the granitoid outcrops. The spectrometer was well-calibrated on artificial concrete pads at the Nuclear Materials Authority of Egypt before the field survey. The pads contain known concentrations of potassium, uranium, and thorium, as described by Grasty et al. [62]. An error propagation equation for systematic and random measurement errors was used to compute the overall uncertainty of the radiation levels. In the efficiency calibration, there were systematic inaccuracies of 0.5 to 2% and random errors of up to 5% in the radioactivity values. Table 1 shows how to calculate the radiological risk parameters using the activity concentrations of ^238^U, ^232^Th, and ^40^K, as well as the mathematical formulas.

### 3.2. Heavy Minerals

In this work, there was an identification of the important minerals in the mineralized zone to the south of the area at an old Roman mine (Wadi Makhrag El Ebel). The collected mineralized samples were separated by the operation of shaking tables using water and heavy liquid (bromoform, 2.8 specific gravity) to separate the light fractions from heavy minerals. Magnetite was separated from the heavy fraction using a hand magnet. The heavy mineral fractions were separated into magnetic and nonmagnetic fractions using a high-intensity, left-type magnetic separator (Carpco, Model MLH (13) 111-5). Then, the heavy minerals were easily picked as individual minerals with a binocular microscope.

The separated minerals were analyzed using an environmental scanning electron microscope (ESEM) supported by an energy dispersive spectrometer (EDS) unit (model Philips XL 30 ESEM) at the laboratories of the ENMA. The analytical conditions were 25–30 Kv accelerating voltages, 1–2 mm beam diameters, and 60–120 s counting times. The minimum detection limit varied from 0.1 to 1 wt%. The precision was well below 1%, and the relative accuracy of the quantitative results was 2–10% for elements with Z > 9 (F) and 10–20% for light elements (B, C, N, O, and F). Copper minerals were also confirmed with an X-ray diffraction (XRD) technique using American Society of Testing Materials (ASTM) cards no. (5-0661) and (5-0490) for tenorite and quartz, respectively.

### 3.3. Statistical Analysis

A Pearson correlation matrix (PCM), a principal component analysis (PCA), and a hierarchical cluster analysis are examples of the MSA used in this study (HCA). These analyses were used to highlight and reveal the relationships between radioactive variables, notably the impact of projected radiological danger factors on the distribution of radionuclide concentrations in beach sand. A PCA was used to discover how strong the connection between the radioactive variables was. PCA is a standard method for summarizing a collection of patterns among the analyzed variables of a dataset and for demonstrating their similarities and differences. For the PCA evaluation, the data were processed using varimax normalized technique. PCA has the advantage of compressing data by reducing the number of dimensions while retaining as much information as feasible. EMB-SPSS Version 21.0 was used for the statistical analysis of the data.

## 4. Results and Discussion

### 4.1. Mineralogical Features of Heavy Minerals

The investigated mineralization associated with monzogranites included radioactive-bearing minerals such as monazite and zircon, as well as copper and barite. On the other hand, gold and pyrite were the most important minerals separated from the quartz samples of the old Roman mine in south Monqul at Wadi Makhrag El Ebel.

#### 4.1.1. Radioactive-Bearing Minerals

**Figure 4:**
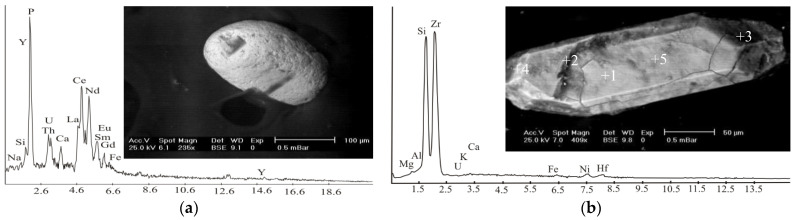
Back-scattered electron (BSE) images and EDS chart for (**a**) monazite; (**b**) zircon from monzogranite at Monqul, north Eastern Desert, Egypt.

*Monazite* is a light, rare, earth phosphate that was determined using an EDS (Figure 4a). Most of the monazite grains were subhedral grains with different shapes, including oval and, sometimes, tabular. Pits and grooves were common features on the surfaces of monazite grains (Figure 4a). The monazite structure could accept REE ions with ionic radii between those of La and Eu. The EDS data showed that monazite was composed of P (26.79 wt%), and its REEs were La, Ce, Nd, Sm, Gd, and Eu (18.53, 32.28, 2.23, 7.54, 1.35, and 0.56 wt%, respectively) (Figure 4a). Monazite grains contained Th (5.85 wt%) with relatively low U (0.83 wt%). Na, Si, Fe, and Y occurred as minor constituents. The Ce showed a marked predominance of REEs, designating monazite-(Ce).

*Zircon* occurs as euhedral prismatic-zoned crystals hosting clusters of opaque microinclusions. Colorless zircon is common; additionally, zircon grains with yellowish dust colors can be found. The most common colors are red and brown. The general brown-yellow color may be due to Fe oxide staining.

The analyses of the studied zircon using the EDS showed that it was composed essentially of Zr and Si with averages of 66.5 and 24.4 wt%, respectively, as depicted in (Table 2) and (Figure 4b). Other elements present in minor amounts were Mg, Al, Ca, K, Fe, and Ni. Hf and U were substituted in small amounts for part of Zr. The pyramidal crystal habit was the main characteristic. The crystal morphology of primary zircon depends on the crystallization rate, temperature, pressure, melt, and trace element composition [63]. The formation of prism faces in zircon has been attributed to the presence of U, Th, Y, and other REEs, as well as P in the Zr sites [40,47,54,64].

#### 4.1.2. Other Accessory Minerals

##### Copper Mineralization

**Figure 5:**
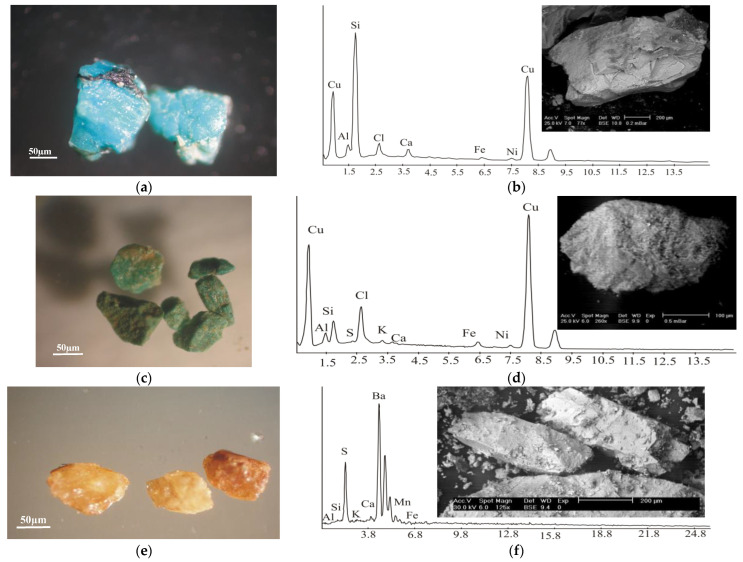
Copper and barite mineralization from monzogranite of Monqul area, north Eastern Desert, Egypt. (**a**) Photomicrographs showing blue grains of chrysocolla under binocular microscope; (**b**) BSE images and EDS chart for chrysocolla; (**c**) photomicrograph showing green grains of tenorite minerals under binocular microscope; (**d**) BSE images and EDS chart for tenorite; (**e**) photomicrograph showing yellowish-brown and brown grains of barite minerals under binocular microscope; and (**f**) BSE images and EDS chart for barite.

Chrysocolla and tenorite (Figure 5a–d) were the copper minerals identified with the EDS technique. Additionally, tenorite was confirmed with an X-ray diffraction (XRD) technique. Copper ore deposits could be extracted through two conjugate normal fault systems in monzogranites trending E–W in the southern part of the Monqul area at Wadi Makhrag El Ebel.

*Chrysocolla* is a mineral of secondary origin commonly associated with other secondary copper minerals, such as cuprite and tenorite. It occurs as a massive, angular earthy aggregate with a botryoidal crust. The most-recorded colors are green, bluish-green, blackish-blue, brown, and blue (Figure 5a,c). Lustrous black botryoidal tenorite mostly covered a sky-blue, massive chrysocolla matrix. The EDS analyses showed that chrysocolla was composed essentially of copper silicates in which Cu had a 54.52 wt% and Si had a 33.82 wt%, with other elements present in minor amounts, such as Al, Cl, Ca, Fe^+3^, and Ni (Figure 5b). 

**Figure 6:**
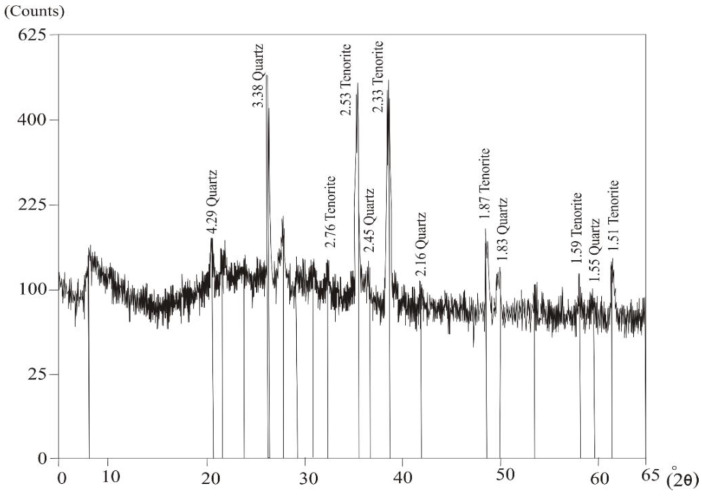
X-ray diffraction (XRD) patterns of tenorite and quartz from monzogranite at Monqul, north Eastern Desert, Egypt.

*Tenorite* is a secondary mineral found in the oxidized zones of copper ore deposits. It is present in the form of an earthy aggregates, ranging from pale green to green (Figure 5c) or steel-grey to black in color when exposed to heating. The EDS analyses of the studied tenorite showed that it was composed essentially of Cu (82.45 wt%) with other elements that were present in minor amounts (Al, Si, S, Cl, K, Ca, Fe^+3^, and Ni) (Figure 5d). Additionally, tenorite was confirmed with an X-ray diffraction (XRD) technique, as shown in (Table 3) and (Figure 6).

On the other hand, Botros and Wetait [65] stated that the most common copper minerals in the mineralized zone of the Monqul area at Wadi Makhrag El Ebel were chalcopyrite, bornite, enargite, and covellite. However, the present study showed that copper minerals were represented mainly by chrysocolla and tenorite.

*Barite* is considered an important commercial mineral and is widely used as a pigment in the preparation of lithopone and as a filter for paper and cloth. Barite mud is poured into deep oil wells to buoy up drilling tools. It is the principal ore of barium, and it is used in paint and as radiation isolated material in the nuclear industry. The barite veins in the Monqul area cut through the younger granite (monzogranite) and range from minute veinlets to a size of 2 m in width. These veins are associated with the silicification of monzogranite in the form of secondary quartz, kaolinitization, and the sericitization of plagioclase.

Barite minerals are fine-to-medium-grained; colorless, white, red or brown; and show a glassy luster (Figure 5e). Barite crystals are massive, granular, earthy aggregate plates. The analyses of the studied barite using EDS (Figure 5f) showed that it consisted essentially of Ba (70.88 wt%) and S (27.22 wt%). Other elements, including Si at 0.53 wt%, Al at 0.31 wt%, Fe at 0.51 wt%, K at 0.08 wt%, Ca at 0.13 wt%, and Mn at 0.34 wt%, were present.

**Figure 7:**
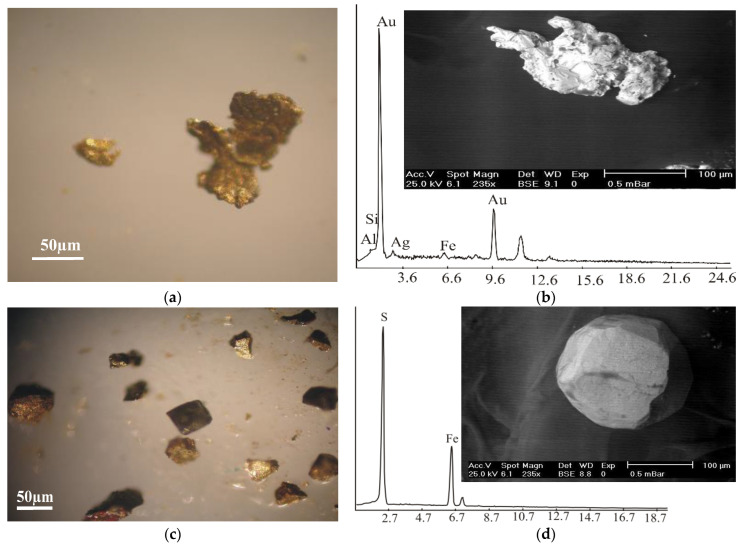
Gold and pyrite mineralization from quartz veins in south Monqul, north Eastern Desert, Egypt. (**a**) Photomicrograph showing yellowish grains of gold under binocular microscope; (**b**) BSE images and EDS chart for gold; (**c**) photomicrograph showing brownish grains of pyrite under binocular microscope; and (**d**) BSE images and EDS chart for pyrite.

*Gold* occurs as disseminated specks associated with sulphides that are altered by goethite [65]. It is also dispersed in barite veins associated with the high sulfidation minerals of enargite, barite, pyrite, and chalcopyrite. The present work dealt with the treatment of tailings and dumps of old work in younger granite in the southern part of Monqul, which had many advantages. The dumps were easily drilled, pitted, and sampled. It can be confused with metal sulphides, but it is distinguished from them by its softness and malleability (Figure 7a). Therefore, the crystal morphology of gold is variable, from plates, wires, and dendrites to compact and cavernous. It is characterized by golden-yellow, yellow, and sometimes shiny black colors (Figure 7a,b). The analyses of the studied gold using EDS showed that the ore was composed essentially of Au (94.18 wt%), with small amounts of Si (0.73 wt%), Al (0.14 wt%), and Fe (1.24 wt%). The analyses showed an amount of Ag at 3.71 wt% associated with gold (Figure 7a,b).

*Pyrite* is used as a source of sulphur for the manufacture of sulphuric acid. It is characterized by its yellow, brass-yellow, and reddish colors due to the effect of oxidation (Figure 7c). It displays a strong tendency towards idiomorphic crystal growth. Therefore, it was found, as well as euhedral crystals of a cubic habit (Figure 7c,d). It is frequently rich in gold values and, therefore, may be an important ore of gold. Pyrite is composed essentially of S (53.27 wt%) and Fe (46.73 wt%). It is formed simultaneously with its associated minerals (rutile, hematite, magnetite, and goethite), but it is distinguished from them by its greater form energy. 

### 4.2. Radiological Implications

#### 4.2.1. ^238^U, ^232^Th, and ^40^K

The ^238^U, ^232^Th, and ^40^K activity concentrations of the radionuclides were monitored in the studied monzogranites and are described in Table 1. The ranges (mean value ± standard deviation) of the activity concentrations for ^238^U, ^232^Th, and ^40^K in the monzogranites were r_U_ 19-564 (103 ± 91), r_Th_ 50-174 78 ± 19), and r_K_ 657-3098 (1484 ± 334) Bq kg^−^^1^, respectively. The natural radioactivity rates vary widely due to variations in the activity concentration values for ^238^U, ^232^Th, and ^40^K in geological formations, as well as their physical, chemical, and geochemical characteristics [66]. The achieved results demonstrated that the mean data of ^238^U, ^232^Th, and ^40^K were three, two, and four times greater than the worldwide data of 33, 45, and 412 Bq kg^−1^, respectively [3]. Weathering, leaching, alteration, and aeration activities may have enhanced radioactive levels in the examined materials. The presence of ^238^U, ^232^Th, and ^40^K in high concentrations in the monzogranite samples was due to the accumulation of radioactive minerals, including thorite and uranothorite, as well as zircon, monazite, allanite, clay, feldspar, etc [67]. Descriptive analyses (skewness, kurtosis, and coefficient variance; %) were performed on the activity concentrations of ^238^U, ^232^Th, and ^40^K and are presented in Table 4.

**Figure 8:**
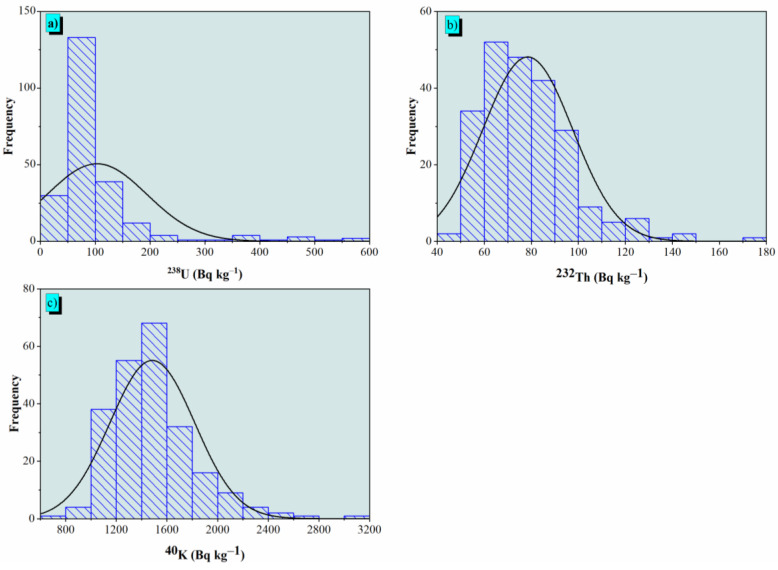
Distribution of the activity concentrations of ^238^U, ^232^Th, and ^40^K in Bq kg^−1^ of the monzogranites in the Monqul area, north Eastern Desert, Egypt.

It was clarified that the standard deviation (SD) values of the radionuclide concentrations were lower than their mean values. This demonstrated a significant amount of symmetry for ^238^U, ^232^Th, and ^40^K in the monzogranite samples. The asymmetry distribution and the peakness of probability were controlled by the skewness and the kurtosis, respectively. It can be clarified in Table 1 that the skewness values of ^238^U, ^232^Th, and ^40^K were positive. Therefore, the natures of ^238^U, ^232^Th, and ^40^K were asymmetric in distribution. Moreover, the positive values of the kurtosis factor illustrated the peak probability distribution. The variation coefficient (CV, %) values were high for ^238^U (88%) and moderate for ^232^Th (24%) and ^40^K (22%). The variation was explained by the monzogranite samples containing radioactive minerals of the radioactive elements. The frequency distributions of the ^238^U, ^232^Th, and ^40^K activity concentrations of the monzogranite samples are shown in Figure 8a–c. 

A normal distribution was achieved for the thorium activity concentration, where the distribution of uranium and potassium was characterized by multimodality. The modality feature was due to the complexity of the heavy radioactive minerals in the monzogranite samples. The normality analysis of ^238^U, ^232^Th, and ^40^K distribution was estimated using IBM-SPSS version 21.0 (a commercial statistics software package) and applied based on the Kolmogorov–Smirnov (KS) test and presented in Table 5.

The KS test depicted the *p*–value of thorium concentration as greater than 5%, revealing that the ^232^Th distribution was normal. The activity concentrations of ^238^U, ^232^Th, and ^40^K in the studied monzogranites were compared with various previous investigations in other countries (see Table 6).

#### 4.2.2. Hazards and Cancer Assessment

The probability of the application of monzogranites in construction materials is governed by the effectiveness of the emitted gamma radiation. Therefore, sundry radiological variables were estimated and are presented in Table 7. The radium equivalent content (Ra_eq_) values alternated from 177 to 971 Bq kg^−1^ with a mean value of 330 Bq kg^−1^, which was lower than the permissible limit of 370 Bq kg^−1^ [76]. This revealed that the monzogranites are convenient to employ in building materials, but this depends on the identification of other radiological factors. High Ra_eq_ values were recorded in 19% of the monzogranite samples. 

The effectiveness of the released gamma photon energy on the public is described by the absorbed dose rate (D_air_) and the annual effective dose (AED_out_ and AED_in_). The D_air_ is predicated by the interaction of the emitted gamma photon energy with the human body 1 m above the ground. Table 7 displays the estimated D_air_, which ranged between 84 and 452 nGy/h with a mean value of 156 nGy/h, which was less than the UNSCEAR recommended value (59 nGy/h) [3]. Thus, the released gamma rays from the monzogranites can affect humans, and exposure to gamma radiation for a long time can pose adverse health effects. This can be confirmed by the annual effective dose values of indoor and outdoor (Table 7). The mean values of AED_out_ and AED_in_ were 0.19 and 0.77 mSv/y, respectively, which were higher than the permissible levels of 0.07 mSv/y (outdoor) and 0.41 (indoor) mSv/y [3]. This implied that long-term exposure to a low rate had no detrimental health consequences, such as tissue deterioration, DNA in the genes, cancer, or cardiovascular disease [77]. The minimum and maximum AED_out_ and AED_in_ values exceeded the permissible level, where the AED_out_ min–max was 0.10–0.55 mSv/y and the AED_in_ min–max was 0.41–2.2 mSv/y. 

Due to exposure to the released gamma radiation from the monzogranites, the ELCR_t_ was observed to be expanded from 1.79 × 10^−6^ to 9.69 × 10^−6^ with a mean value of 3.35 × 10^−6^. The mean value was much higher than the permissible limit of 0.29 × 10^−6^ [78]. Low doses of radiation have been shown to improve human health by speeding up the DNA repair process, lowering genetic fragility, and improving overall immunological response [79,80]. It also helps treat inflammation of the lymph nodes, relieve arthritis, heal wounds, and treat infections [81].

**Figure 9:**
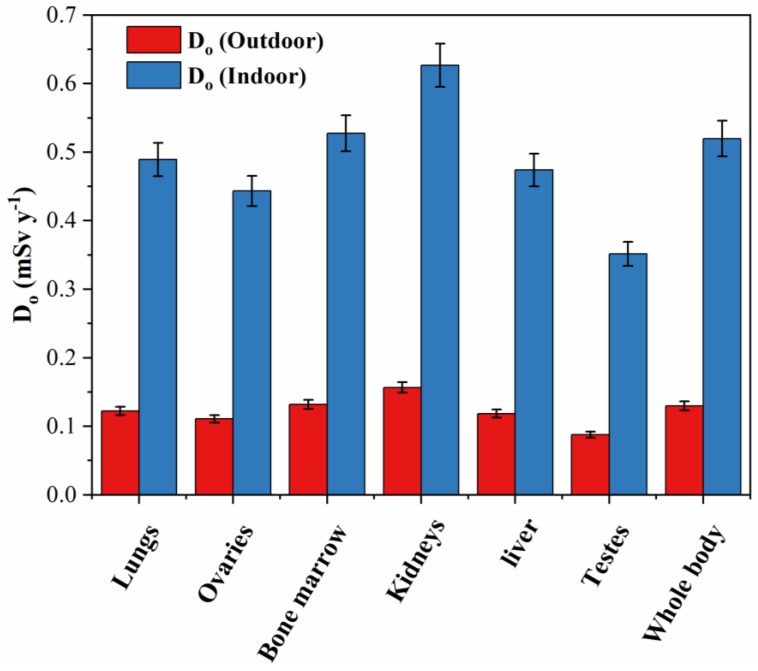
The D_o_ (mSv y^−1^) associated with tissues from outdoor and indoor air doses of the monzogranites.

The D_o_ computes how much radiation is retained in different human organs over a year of exposure, as shown in Figure 9. Indoor gamma radiation exposure yielded the highest D_o_ mean values for all the examined organs, ranging from 0.35 mSv/y (liver) to 0.63 mSv/y (testes), while the D_o_ were predicted with low values for outdoor exposure to gamma radiation and varied from 0.09 mSv/y (liver) to 0.16 mSv/y (testes). The registered results of D_o_ illustrated that the mean values were lower than the permissible threshold dose of 1.0 mSv/y. The liver contributed the lowest dose due to the rate of absorption of nutrients from the diet [82]. This indicated that monzogranites in the studied area do not have a significant effect on the radiation dosage received by these adult tissues.

### 4.3. Statistical Analysis

#### 4.3.1. Pearson Correlation Analysis (PC)

Pearson correlation (PC) was used in this investigation to reveal strong links and linear relationships between ^238^U, ^232^Th, and ^40^K activity concentrations and radiological danger indicators in the monzogranite samples. The PC analysis describes the correlation strength of linear relations, which it divides into four groups: weak (0.3–0.49), moderate (0.5–0.69), strong (0.7–0.9), and very strong (>0.9) correlations [83]. The correlations between all the radiological parameters were positive (see Table 8). Weak correlations were observed between ^238^U with ^232^Th (R^2^ = 0.34) and a moderate correlation with ^40^K (R^2^ = 0.54). This was explained by the investigated monzogranites enriched with uranium and potassium minerals. It can be observed in Table 5 that the correlations of ^238^U and ^40^K with the radiological variables were very strong and strong, respectively. Therefore, uranium and potassium were considered the main contributors to adverse health effects linked to the released gamma radiation from monzogranite. 

#### 4.3.2. Hierarchical Cluster Analysis

**Figure 10:**
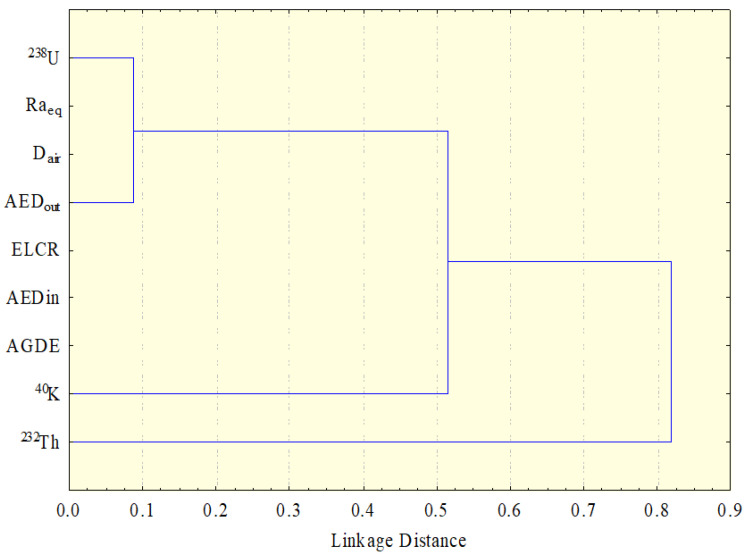
Dendrogram showing the clustering of variables.

Due to a large number of parameters, correlation analysis data were deemed difficult. Nevertheless, hierarchical cluster analysis was used to uncover and expose the correlations between the radioactive properties informally (HCA). HCA is a data classification system that identifies real-world data types using multivariate algorithms. Objects are grouped in such a way that they all belong to the same category. The data with the highest level of similarity are grouped first, followed by the data with the next best fit in hierarchical clustering. This process is repeated until all of the data have been classified. The degrees of similarity between the data are used to generate a dendrogram. A similarity of 100 percent means that the clusters are separated by zero distance from comparable sample measures, whereas a similarity of 0 percent suggests that the clustering areas are as dissimilar as the least similar location. In this work, Ward’s technique was employed to perform cluster analysis. Ward’s method connects radioactive activity concentrations with radiological variables to calculate their Euclidean distances [84]. Figure 10 displays the dendrogram classified into two main clusters of the examined data. Cluster I contains ^238^U, ^40^K, and the radiological parameters, while Cluster II contains the rest of the radiological parameters and ^40^K. The HCA defined the radioactivity of granite as being linked to the activity of radioactive concentrations, especially radium and thorium. The HCA data of the Pearson correlation were observed to correspond.

#### 4.3.3. Principal Component Analysis (PCA)

**Figure 11:**
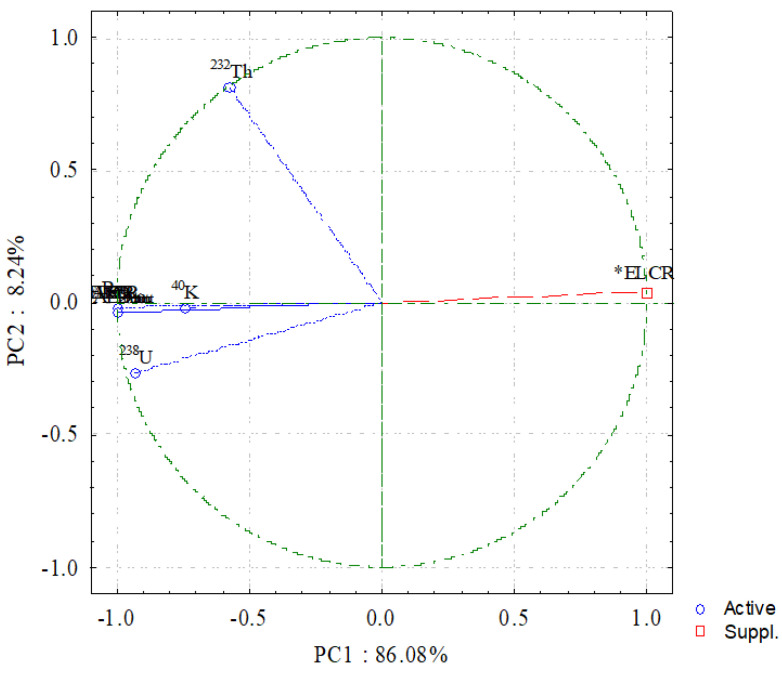
Graphical representation of PC1 and PC2; * is supplementary variable.

A PCA was utilized to find the matrix correlations between multiple components using varimax rotations in this study. Figure 11 demonstrates the PC1 and PC2 components. As a result, ^238^U and ^40^K were the most common gamma-emitting sources in all the monzogranites in the study area. Furthermore, as previously stated, the mineral analysis revealed the presence of radioactive-carrying minerals in the studied monzogranites. An amount of 86.08% of the variation can be explained. As a result, ^238^U and ^40^K were the predominant natural radioactive contributors to the monzogranite in the study area. ^232^Th, on the other hand, displayed a negative loading in the PC2 load. The amount of variance that could be explained was 8.24%. The loading contrast that thorium saw did not affect the exposure phase. According to the PC analysis, the overall explained variance in the radioactive data was 94.32 percent; as a result, the data appeared to be good [85,86]. 

## 5. Conclusions

The monzogranite of Wadi Makhrag El Ebel was influenced by substantial hydrothermal alteration with copper minerals (chrysocolla and tenorite) and was restricted to argillic, phyllic, and propylitic alterations. The NE–SW and ENE–WSW patterns were seen in gold-bearing quartz and barite veins, respectively. The monzogranites were rich in heavy, radioactive-bearing minerals, particularly monazite and zircon, which were responsible for the increased radioactivity in the south Monqul area. The acquired results revealed that the activity concentration means of 238U, 232Th, and 40K were 103 ± 91, 78 ± 19, and 1484 ± 334 Bq kg^−1^, respectively, which were greater than the allowed global values of 33, 45, and 412 Bq kg^−1^. The radium equivalent content, the absorbed dose rate, the annual effective dose, and many other radiological hazard factors were evaluated. Furthermore, the mean values of lifetime cancer risk (3.35 × 10^−6^) were calculated. The radioactivity of monzogranites was mostly contributed to and dominated by ^238^U and ^40^K according to multivariate statistical techniques. Zircon, monazite, thorite, uranothorite, and other radioactive minerals were found in the granites. As a result, the Monqul granites are one of Egypt’s highest U favorability areas, and employing the granites in building materials is a public health risk.

## Figures and Tables

**Table 1:** Important radiological parameters and indices.

Parameter	Symbol	Definition	Formula
Radium equivalent activity	Ra_eq_	Radium equivalent activity is a weighted sum of the ^226^Ra, ^232^Th, and ^40^K activities according to the hypothesis that 370 Bq kg^−1^ of ^226^Ra, 259 Bq/kg of ^232^Th, and 4810 Bq/kg of ^40^K attain the same dose rates of gamma rays.	Ra_eq_ (Bq kg^−1^) = A_Ra_ + 1.43 A_Th_ + 0.077 A_K_
Absorbed dose rate	D (nGy/h)	The absorbed dose rate is the radioactive factor that is applied to detect the effect of gamma radiation at 1 m from the radiation source in the air due to the concentrations of ^238^U, ^232^Th, and ^40^K.	D_air_ (nGy h^−1^) = 0.430 A_U_ + 0.666 A_Th_ + 0.042 A_K_
Outdoor annual effective dose	AEDout	The annual effective dose is a radioactive factor utilized to detect the exposure level of radiation during a stationary duration (1 year).	AED_out_ (mSv/y) = D_air_ (nGy/h) × 0.2 × 8760 (h/y) × 0.7 (Sv/Gy) × 10^−6^ (mSv/nGy)
Indoor annual effective dose	AEDin		AED_in_ (mSv/y) = D_air_ (nGy/h) × 0.8 × 8760 (h/y) × 0.7 (Sv/Gy) × 10^−6^ (mSv/nGy)
Excess Lifetime Cancer Risk	ELCR	Excess lifetime cancer risk is the radioactive factor applied to detect fatal cancer resulting from gamma radiation exposure at a set duration time (DL = 70 years for public) and risk factor (RF = 0.05 Sv^−1^).	ELCR = AED_out_ × DL × RF
Effective dose to various body organs	D_o_	The computation of Do values is dependent on the scenario of exposure (outdoor—AED_out_—and indoor—AED_in_). The differentiation of effective doses among organs is governed by the dose conversion factor (F). The F values are estimated by the ICRP for each organ (F: 0.46—liver, 0.58—ovaries, 0.62—kidneys, 0.64—lungs, 0.69—bone marrow, 0.82—testes, and 0.68—whole body).	D_o_ (mSvy^−1^) = AED × F

**Table 2:** The semiquantitative results (oxides, wt%) of EDS analyses of zircon grains from monzogranite at Monqul, north Eastern Desert, Egypt.

Spot No.	Zr1	Zr2	Zr3	Zr4	Zr5
Si	29.81	21.85	21.80	23.43	25.55
Al	n.d.	3.74	4.15	1.60	0.78
Fe	0.76	4.96	3.49	0.69	0.64
Mg	n.d.	2.16	2.96	0.05	0.06
Ca	n.d.	0.40	n.d.	0.25	n.d.
K	n.d.	n.d.	n.d.	0.55	n.d.
Hf	1.90	1.29	2.74	3.19	2.59
Zr	67.53	64.45	64.41	67.36	68.75
U	n.d.	n.d.	0.46	0.60	n.d.
Ni	n.d.	1.15	n.d.	2.29	1.63
Total	100				

**Table 3:** Identification of tenorite and associated quartz from monzogranite using X-ray diffraction (XRD) technique at Monqul, north Eastern Desert, Egypt.

Analyzed Samples		Identified Minerals			
		Tenorite		Quartz	
		(ASTM) Card No.			
		(5-0661)		(5-0490)	
dÅ	I/I_0_	dÅ	I/I_0_	dÅ	I/I_0_
4.29	18			4.26	35
3.38	100			3.343	100
2.76	10	2.751	12		
2.53	97	2.523	100		
2.45	9			2.458	12
2.33	93	2.323	96		
2.16	4			2.128	9
1.87	21	1.866	25		
1.83	11			1.817	17
1.59	7	1.581	14		
1.55	3			1.541	15
1.51	14	1.505	20		

**Table 4:** Activity concentrations of ^238^U, ^232^Th, and ^40^K (Bq kg^−1^) in the monzogranites and the descriptive statistics.

	N	Mean	SD	Min	Max	Skewness	Kurtosis	CV, %
U-238	231	103	91	19	564	3.22	11.20	88
Th-232	231	78	19	50	174	1.35	3.20	24
K-40	231	1484	334	657	3098	1.15	2.67	22

**Table 5:** Normality test using Kolmogorov–Smirnov.

	DF	Statistic	*p*-Value
U-238	231	0.26	6 × 10^−14^
Th-232	231	0.08	0.10
K-40	231	0.10	0.02

**Table 6:** Comparison of ^238^U, ^232^Th, and ^40^K activity concentrations in the Monqul area with numerous world studies.

Country	^238^U	^232^Th	^40^K	References
Spain	84	42	1138	[68]
Iran	77.4	44.5	1017.2	[69]
India	25.88	42.82	560.6	[70]
Jordan	41.52	58.42	897	[71]
Palestine	71	82	780	[72]
Egypt	137	82	1082	[73]
Greece	74	85	881	[74]
Turkey	80	101	974	[75]
Saudi Arabia	28.82	34.83	665.08	[76]
Egypt	103	78	1484	Present study

**Table 7:** The associated environmental hazard variables in the monzogranites.

	Ra_eq_	D_air_	AED_out_	AED_in_	ELCR_out_ × 10^−6^	ELCR_in_ × 10^−6^	ELCR_t_ × 10^−6^
Mean ± SD	330 ± 120	156 ± 56	0.19 ± 0.07	0.77 ± 0.28	0.67 ± 0.24	2.98 ± 0.96	3.35 ± 1.21
Range	177–971	84–452	0.10–0.55	0.41–2.22	0.36–1.94	1.43–7.75	1.79–9.69

**Table 8:** Pearson correlation matrix for variables.

	U-238	Th-232	K-40	Ra_eq_	D_air_	AED_out_	AED_in_	ELCR
U-238	1							
Th-232	0.34	1						
K-40	0.54	0.37	1					
Ra_eq_	0.95	0.56	0.71	1				
D_air_	0.95	0.55	0.73	0.99	1			
AED_out_	0.95	0.55	0.73	0.99	0.99	1		
AED_in_	0.95	0.55	0.73	0.99	0.99	0.99	1	
ELCR	0.95	0.55	0.73	0.99	0.99	0.99	0.99	1

## Data Availability

Data are contained within the article.
